# Odor Experience Facilitates Sparse Representations of New Odors in a Large-Scale Olfactory Bulb Model

**DOI:** 10.3389/fnana.2016.00010

**Published:** 2016-02-11

**Authors:** Shanglin Zhou, Michele Migliore, Yuguo Yu

**Affiliations:** ^1^School of Life Science and The Collaborative Innovation Center for Brain Science, The Center for Computational Systems Biology, Fudan UniversityShanghai, China; ^2^Division of Palermo, Institute of Biophysics, National Research CouncilPalermo, Italy; ^3^Department of Neurobiology, Yale University School of MedicineNew Haven, CT, USA

**Keywords:** odor representation, prior experience, sparse representation, olfactory bulb, large scale network

## Abstract

Prior odor experience has a profound effect on the coding of new odor inputs by animals. The olfactory bulb, the first relay of the olfactory pathway, can substantially shape the representations of odor inputs. How prior odor experience affects the representation of new odor inputs in olfactory bulb and its underlying network mechanism are still unclear. Here we carried out a series of simulations based on a large-scale realistic mitral-granule network model and found that prior odor experience not only accelerated formation of the network, but it also significantly strengthened sparse responses in the mitral cell network while decreasing sparse responses in the granule cell network. This modulation of sparse representations may be due to the increase of inhibitory synaptic weights. Correlations among mitral cells within the network and correlations between mitral network responses to different odors decreased gradually when the number of prior training odors was increased, resulting in a greater decorrelation of the bulb representations of input odors. Based on these findings, we conclude that the degree of prior odor experience facilitates degrees of sparse representations of new odors by the mitral cell network through experience-enhanced inhibition mechanism.

## Introduction

Prior sensory experience is very important for animals in learning and processing novel incoming signals. In olfaction, prior odor experience can significantly improve the ability of the animal to discriminate new odor inputs (Mandairon et al., 2006a,b,c; Mandairon and Linster, 2009; Sinding et al., 2011). The olfactory bulb is the first relay of the olfactory pathway, and encodes odor inputs as the network responses of mitral cells (Kay and Sherman, 2007; Mandairon and Linster, 2009). The olfactory bulb has been observed to encode signals in a spatiotemporally sparse and decorrelated manner (Khan et al., 2010; Yu et al., 2014). Moreover, it has been observed that mitral cells become less responsive after prior odor exposure (Buonviso et al., 1998; Buonviso and Chaput, 2000; Fletcher and Wilson, 2003; Mandairon and Linster, 2009; Kato et al., 2012). On the other hand, it has been shown that interneurons may become more (Mandairon et al., 2008) or less (Kato et al., 2012) responsive with new odors.

In previous experimental and computational studies, the numbers of prior odor experiences and new incoming odors are limited. How an animal's prior experience with odorants affects the representation by the olfactory bulb (i.e., the firing patterns of mitral and granule cells) in response to new odors is an open question. Considering the limitations of current experimental techniques, it is nearly impossible to access the synaptic dynamics or neuronal response to odor inputs in the olfactory bulb network at a large scale. However, large-scale supercomputer simulation of realistic olfactory bulb models has been employed to carry out a series of simulations examining these issues (Yu et al., 2013, 2014; Migliore et al., 2014, 2015). Our previous reports have shown that a sparse spatial spiking representation of specific odor signals can emerge naturally from mitral-granule interactions and can be realistically implemented by our model via balanced excitatory-inhibitory synapses (Yu et al., 2013, 2014). Here, we examine how and to what extent prior odor experience modulates the excitatory and inhibitory interactions and how they shape odor representations.

To address these issues, we performed a series of simulations based on a previously established large-scale olfactory bulb model (Yu et al., 2013, 2014). The simulation results show that prior odor experience can accelerate the formation of sparseness in the mitral cell network in response to new odors. Furthermore, the sparseness of the mitral cell network is increased but the sparseness of the granule cell network is decreased with an increasing number of prior training odors. Further analysis demonstrated that this phenomenon is accompanied by a nonlinear change in the excitatory and inhibitory synaptic weighting of the network. Mitral cell network responses demonstrated a gradual increase in their intrinsic decorrelation property, suggesting an increased odor discrimination ability.

## Material and methods

### Computational simulations

All simulations were carried out with the NEURON simulation program v7.3 (Hines and Carnevale, 1997, 2001) on a Cray XC30 system (INCF, Sweden). All the present work was based on a previously verified scaled-up olfactory bulb model (Yu et al., 2013, 2014). Briefly, The network was composed of multi-compartment canonical models of 500 mitral and 10,000 granule cells, implemented as described in our previous studies (Migliore and Shepherd, 2008; Migliore et al., 2010). The model uses a reduced number of MCs and granule cells (glom: MC: GC = 1: 5: 100). As we have already explained in detail in a previous paper (Yu et al., 2013), the reason for this choice is that our main aim with this model is to understand the basic processes underlying lateral and feedback inhibition in a network. To this purpose the full number of cells is not needed, especially in the presence of experimental data limited to a very small subset of glomeruli; however, the relative ratio between mitral and granule cells is consistent with experimental estimations, validated against a number of experimental findings (Willhite et al., 2006; Shusterman et al., 2011). The canonical model for mitral cells was implemented with 312 compartments representing an axon, the soma, the apical dendrite, and two lateral dendrites each 1.5 mm in length, in the range indicated by anatomical measurements (Mori et al., 1981). Real mitral cells have a number of lateral dendrites that cover a relatively large, bidimensional surrounding area. From this point of view, our simplifying choice of using only two lateral dendrites per mitral cell has the obvious limitation that, since many glomeruli are at variable distances from the single projection tract, the interactions between mitral cells belonging to specific neighboring glomeruli are not precisely represented. However, our additional choice to project the glomeruli into a single tract, results in the interactions of a given mitral cell with many nearby mitral cells still holding in a generic sense, so that the model gives a relatively accurate reflection of these population interactions within the mitral-granule network. In this way we were able to maintain the requirements for computational resources within a reasonable limit. An indirect proof of the overall quality of this model is its qualitative agreement with a number of experimental findings (Yu et al., 2013). Uniform passive properties were used, with R_a_ = 150 Ω·cm, τ_m_ = 20 ms, and R_m_ and C_m_ adjusted to obtain an input resistance of about 100 MΩ. Resting potential was set at -65 mV and temperature at 35°C. Cells were modeled as regular firing cells (Migliore et al., 2005), with Na, KA, and KDR conductances uniformly distributed over the entire dendritic tree (Bischofberger and Jonas, 1997). Kinetics for the Na conductance were from hippocampal pyramidal neurons (Migliore et al., 1999), whereas those for KA and KDR were from mitral cell data (Wang et al., 1996). Granule cells were modeled with a soma and a 20 segment radial dendrite (250 μm of total length) representing the dendritic tree. Na^+^ and KA channels were distributed throughout (Schoppa and Westbrook, 1999; Pinato and Midtgaard, 2005; Zelles et al., 2006) whereas KDR was present only in the soma (Schoppa and Westbrook, 1999).

Effective dendrodendritic coupling between granule cell synapses and mitral cell secondary dendrites was implemented by connecting a GC synapse, containing the same proportion of AMPA and NMDA channels, with the appropriate compartment of mitral cell GABA channel-containing secondary dendrites. The details of the synaptic mechanisms have been described in our previous work (Yu et al., 2013, 2014). It should be noted that we applied a generic use-dependent plasticity rule to the dendrodendritic connection. Briefly, all synaptic weights started at zero and, in response to an odor input, the components (inhibitory or excitatory) of each dendrodendritic synapse were independently modified according to local spiking activity in the lateral dendrite of the mitral cell or the granule cell synapse. After each spike, the peak conductance (*w*) and the state (*p*) of any given synapse were updated from their current value *w*_{*exc, inh*}, *p*_ = *g*_*max*, {*exc, inh*}·*S*(*p*)_ to a new value. The new values were calculated according to the instantaneous presynaptic interspike interval (ISI) (see Migliore et al., 2007) as *w*_{*exc, inh*}, *p* + Δ_ = *g*_*max*, {*exc, inh*}·*S*(*p* + Δ_). The value of *p* was limited to the range 0–50, and is subjected to the classical scheme Δ = {0, +1, −1} (Stanton, 1996) in which Δ = 0 for an ISI ≥ 250 ms (i.e., no changes for spike rates ≤ 4 Hz), Δ = −1 for 33 < ISI < 250 ms (LTD in the range of 4–30 Hz), and Δ = 1 for ISI ≤ 33 ms (LTP for a spike rate ≥ 30 Hz). The sigmoidal activation function *S*(*p*) was defined as S (*p*) = 1/{1 + *exp*[(25**-p**)/3]} (Haykin, 1994). In this way, the weight (i.e., the peak synaptic conductance) of any given synapse could transition from a fully depressed (*w* ≈ 0, for *p* = 0) to a fully potentiated state (*w* ≈ *g*_*max*_, for *p* = 50), or vice-versa, over a span of 50 consecutive spikes of the appropriate frequency. At the beginning of a simulation *p* = 0, the spikes resulting in values of *p* < 0 or > 50 were ignored.

It should be stressed that synaptic plasticity is fundamental to any dynamic network. Although in the mitral-granule circuit it has not been observed directly, we consider this lack of information as a shortcoming of the experimental techniques rather than a demonstration that there is no plasticity in the olfactory bulb. Indeed, recent studies have shown more or less direct evidence for long term plasticity of olfactory input in mitral cells (Ennis et al., 1998; Ma et al., 2012), and in granule cells (Patneau and Stripling, 1992; Gao and Strowbridge, 2009; Arenkiel et al., 2011). Also note that the plasticity rule used in this model has already been shown (Yu et al., 2013) to generate synaptic clusters and firing patterns in qualitative agreement with experimental findings. As discussed in detail elsewhere (Xiong and Chen, 2002; Migliore and Shepherd, 2008), the formation of synaptic clusters consistent with those observed experimentally is an extremely robust process that can be understood by considering the follow dynamics: (a) a strong odor input causes mitral cells to fire at high-frequency; (b) somatic APs backpropagate along the lateral dendrites and potentiate excitatory mitral–granule synapses along their way, activating granule cells; (c) granule cells begin to fire at high-frequency, potentiating inhibitory synapses on the lateral dendrites of mitral cells, (d) inhibition from granule cells hinders AP back-propagation as it travels far from the soma, thus reducing, locally, the firing frequency of mitral and granule cells, and (e) this finally results in the selective depression of synapses far from the soma of the active mitral cell. Therefore, as long as: (1) action potentials backpropagate along the mitral cell lateral dendrites, (2) granule cells form dendrodendritic connections, and (3) LTD and LTP are induced by different levels of synaptic activity, a column will form independently from the specific learning rule. This mechanism is robust and independent of the plasticity rule used to update the synaptic weights during a simulation (Migliore et al., 2007, 2010); we have tested it with hebbian, non-hebbian, and spike-time-dependent plasticity, obtaining in all cases the same qualitative result (i.e., the formation of a column).

It should be noted that in this paper we were interested in the results obtained for a relatively high odor concentration, which is needed to form glomerular units as observed in the experiments. The overall amount of LTP or LTD obtained in a real system, and its overall effect on the I/O properties, will of course depend from the actual plasticity rules in effect for mitral and granule cells. There are no sufficient experimental indications on these processes. However, we stress that the plasticity rule used in this model has already been shown (Yu et al., 2013) to generate synaptic clusters and firing patterns in qualitative agreement with experimental findings.

Other details of the model were identical to those described previously (Yu et al., 2013, 2014). The simulation codes used to run the simulations described in the present work are available in the ModelDB database (http://senselab.med.yale.edu/modeldb, accession number 144570), with the exception of run control files. Kinetic equations and implementation details for all ionic currents are described in these model files.

### Odor input paradigm

In our model, the network contains 100 glomeruli, 500 mitral cells, and 10,000 granule cells. The 100 glomeruli spatially distributed within which 74 glomeruli are chosen to have active responses to represent the spatial responses to 72 different odor stimuli. Each glomerulus makes synaptic connections with five mitral cells. For those 74 glomeruli, there are 370 spatially distributed mitral cells connected to them. The other 130 mitral cells are connected to other 26 glomeruli (which could be stimulated by new odors, other than the present 72 odors). We distributed them in such a way to have a roughly uniform overall spatial distribution of glomeruli. Note that although there is no odor input feeding to those 130 mitral cells, their firing is modulated by the random background activity and by the lateral inhibition received from granule cells that are connected with odor-activated mitral cells. As described in our previous work, 72 odor inputs were used for simulations (Yu et al., 2013, 2014). The basic activation strength (0–4) for each glomerulus and each odor is taken directly from the experimental values kindly provided by Mori et al. (2006). To simulate an odor presentation, these values are multiplied by a coefficient representing the odor concentration, and that resulted in an aggregate synaptic input up to 10 nS, as explained in details in the Methods section of Yu et al. (2013).

A new model of the olfactory bulb, representing the actual 3D layout of the mitral-granule cell network, has been recently developed (e.g., Migliore et al., 2015). This model represents in a very realistic way the possible interactions between glomeruli located within the dendritic field of mitral cells, and it would be especially useful to study natural odors, which exhibit a rather broad and dense input. However, it requires large computational resources. With the particular set of inputs we are considering in this paper, i.e., single monomolecular odors with rather sparse and segregated inputs, such a model would not give results qualitatively different from those obtained with the 1D model.

**Figure 1:**
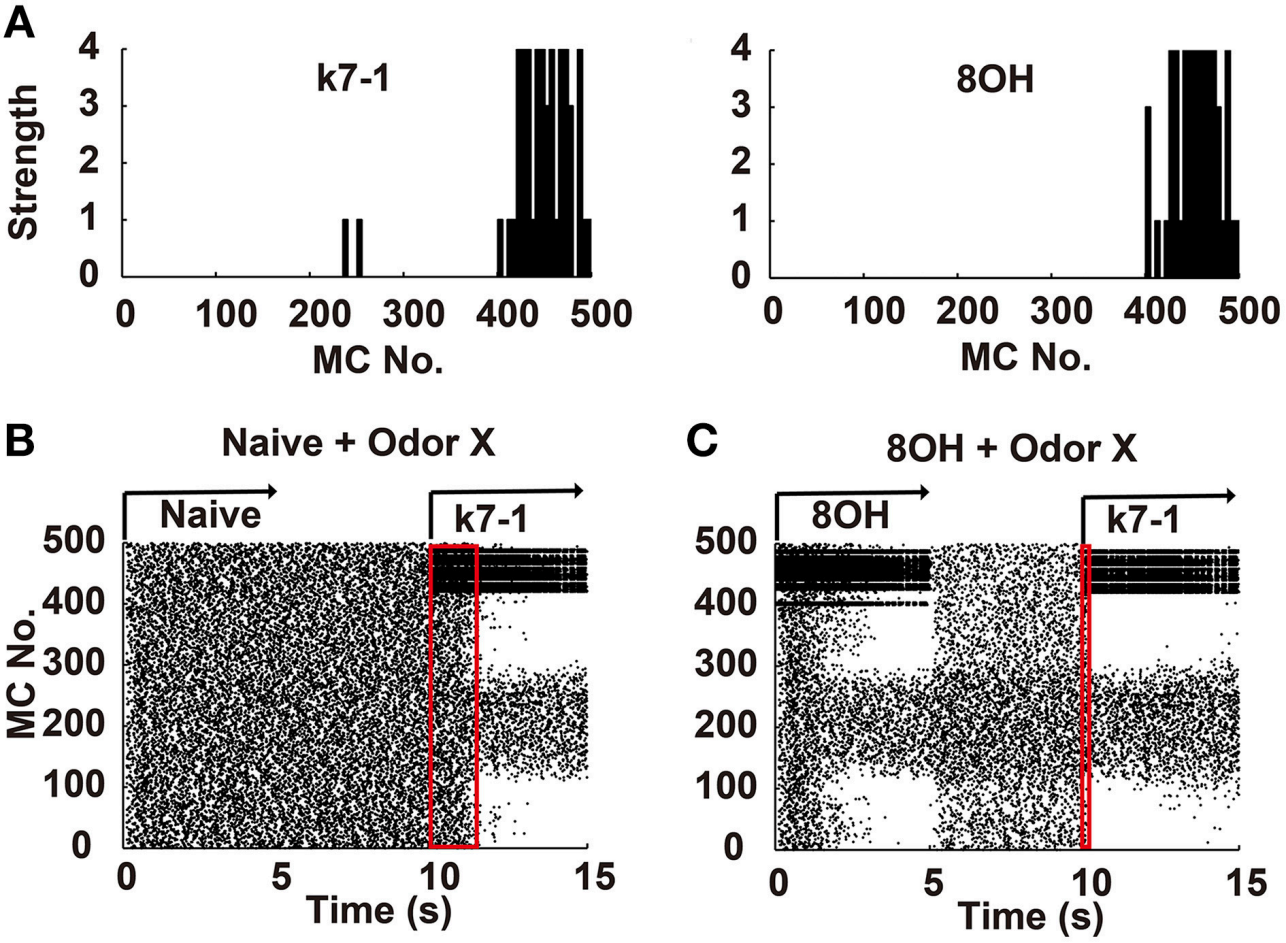
**Mitral cell network responses in naïve and prior odor experiences conditions. (A)** Two example odor input strengths to each mitral cell. k7-1, heptyl methyl ketone; 8OH, octanol. **(B)** A raster plot shows the mitral cell network response in the naïve condition. k7-1 was delivered at the 10th second after a resting state (each mitral cell fires spontaneously and randomly at a low frequency) of 5 s. **(C)** A raster plot shows the mitral cell network response to the new odor input k7-1 in the single odor input (8OH) experience condition. Red rectangles represent the time elapsed to reach a stable sparseness level for the network response in different conditions.

To represent the range of intensities with adequate sensitivity (i.e., including the weakest concentration without saturating the network at the highest concentration), we set the peak conductance sensitivity to give suprathreshold responses to levels 3 and 4. Then, we defined strong inputs as strengths of 3 or 4 and weak inputs as strengths of 0, 1, or 2 (Figure 1A). All odor inputs were presented over 4–10 Hz. To address how prior odor experience interferes with the subsequent sparse representation of new odors, a series of odor inputs was used to train the network in sequence. In one odor experience condition, the first odors were presented within the first 5 s, and after a 5 s rest, another odor input was presented for the next 5 s (Figure 1C). In other experience conditions (for instance, five odors experience), more odor series were presented similarly to the single odor experience condition: each odor input was presented for 5 s, with a 5 s resting state between each presentation. The last odor input was denoted as the new odor input, and all prior odor inputs were defined as experienced odors, implying that in the five odors experience condition, a total of six odor inputs were used. For the control condition, only the new odor inputs were given at the time when the new odors were given in the experience conditions (Figure 1B). Unless otherwise noted, all experienced odors during training were presented in order from low level to high level of input strength.

### Sparseness calculation

The method for the sparseness calculation of network response was identical to our previous work (Yu et al., 2014). Briefly, based on previous work (Vinje and Gallant, 2000; Franco et al., 2007), the sparseness of response to a given stimulus can be calculated as follows:

$$\begin{array}{l} S=\left\{1-\frac{{\left[\overset{N}{\underset{i=1}{\sum }}\left(\frac{r_{i}}{N}\right)\right]}^{2}}{\overset{N}{\underset{i=1}{\sum }}\frac{r_{i}^{2}}{N}}\right\}/\left(1-\frac{1}{N}\right), \end{array}$$

where *S* is the sparseness of the network in one period of odor input (from the beginning of one input to the beginning of the next odor input); *r*_*i*_ is the mean firing rate of mitral cell *i* in that period; *N* is the total number of mitral cell (500). A high sparseness value in our present work indicates only a few neurons with high firing rates.

### Correlation between mitral cell firing in a network

To calculate the correlation between mitral cell firing in a network, we used a coherence measure based on the normalized cross-correlation of neuronal pairs in the network. The coherence between two mitral cell *i* and *j* was measured by their cross-correlation of spike trains at zero time lag within a time bin of τ. Precisely, supposing that a long time interval T (one period of odor input) was divided into small bins of τ, and that two spike trains (value of 0 or 1) were given by *X*(*l*), *Y*(*l*), with *l* = 1, 2, …*K* (here *T/K* = τ), respectively, a coherence for the pair (*K*_*ij*_) was calculated as follows (Wang and Buzsaki, 1996; Yu et al., 2014):

$$\begin{array}{l} K_{ij}\left(\tau \right)=\frac{\overset{K}{\underset{l=1}{\sum }}X\left(l\right)Y\left(l\right)}{\sqrt{\overset{K}{\underset{l=1}{\sum }}X\left(l\right)}\sqrt{\overset{K}{\underset{l=1}{\sum }}Y\left(l\right)}}. \end{array}$$

And then, the correlation between mitral cells across the whole network *K* was defined by the average of *K*_*i, j*_(τ) over all pairs of mitral cells in the network. That is

$$\begin{array}{l} K=\frac{1}{N\left(N-1\right)}\overset{N}{\underset{i=1}{\sum }}\overset{N}{\underset{j=1,j\neq i}{\sum }}K_{ij}\left(\tau \right), \end{array}$$

where *N* is the total number of the mitral cells in the network. And in our present work, τ was taken as 20 ms through the whole analysis.

### Correlation between mitral cell network responses

To compare the similarity between mitral cell network response to odor inputs *x* and *y* during an odor input period, we defined and calculated it as the correlation coefficient (*C*_*xy*_) as follows:

$$\begin{array}{l} C_{xy}=\frac{1}{N}\overset{N}{\underset{i=1}{\sum }}Corrcoef\left\{MC_{i}\left[x\left(t\right)\right],MC_{i}\left[y\left(t\right)\right]\right\}, \end{array}$$

where *MC*_*i*_ is the i'th mitral cell; *MC*_*i*_[*x*(t)] and *MC*_*i*_[y(t)] are the mitral cell network response in an odor input period to odor input *x(t)* and *y(t)* respectively; *Corrcoef* is to calculate the classic correlation coefficient. To investigate how prior odor experience affects the network response to the news odor inputs, we calculated the average of *C*_*xy*_ between one new odor input in the experience conditions and the other tested new odor inputs in the naïve condition.

### 1/2 time of sparseness

To test the dynamic evolution of the sparseness of the mitral cell network response, sparseness values were calculated at series time points when the odor inputs were presented. This sparseness time series could be fitted by the classic logarithmic function as follows:

$$\begin{array}{l} S=A_{2}+\left(A_{1}-A_{2}\right)/\left(1+{\left(\frac{x}{x_{0}}\right)}^{S_{1/2}}\right), \end{array}$$

where *S* is the sparseness, and *S*_1/2_ is the time at which *S* reaches the half of the maximum *S* (*A1*).

### Correlation of input strengths between different odors

In some simulations, we quantified the similarity of two odor inputs by calculating the Pearson correlation coefficient based on their strength values for 500 mitral cells (i.e., 500 values for each odor). A higher correlation coefficient indicates that a pair of odors is more similar.

Data were presented as mean ± SEM. Statistical significance was assessed by paired Student's *t*-test or ANOVA analysis with Tukey's multiple comparison test, and *p* < 0.05 was considered significant. Data analyses were performed using Graphpad Prism software *Version 6.0* (San Diego, USA).

## Results

To systematically address how prior odor experience affects the representation of new odor inputs by the olfactory bulb network, we used a previously verified olfactory bulb network model that includes 500 mitral cells and 10,000 granule cells connected through dendrodendritic synapses (Yu et al., 2013, 2014). In this model, we simulated different odor inputs to mitral cells with varied strength intensities ranging from 0 to 4 based on previous experimental results (Mori et al., 2006; Figure 1A). As shown in Figure 1B, in the naïve condition (i.e., no prior odor input experience, without odor inputs during the first 10 s), a sparse spatial spiking representation of specific odor input (k7-1 in this example) emerged naturally within several seconds of the training period from the mitral-granule cell interactions, as verified by our previous work (Yu et al., 2013, 2014). In one training paradigm, after delivery of a prior odor input (8OH) for 5 s and a 5 s resting state (no odor input), a new odor input (K7-1or other, see below) induced a different response of mitral cell network compared with that observed in the naïve condition (Figure 1C, compare with the mitral cell network response during the period of 10th–15th second in Figure 1B). From the raster plot, we observed that the response of the mitral cell network reached a stable sparseness state much faster than the naïve condition (Figures 1B,C, note that the red rectangle denotes the course to reach stable sparseness in Figure 1C that is much narrower than in Figure 1B). Since the sparseness of the mitral cell network reaches steady state after about 2 s of odor stimulus, we trained the network with specific odor input for 5 s in the following results. We also extended the simulation time to 10 s, and no significantly different results were found (Supplementary Figure 1). We will now present additional details describing our results.

### Prior odor input experience facilitates the evolution of the sparseness of the mitral cell network response

**Figure 2:**
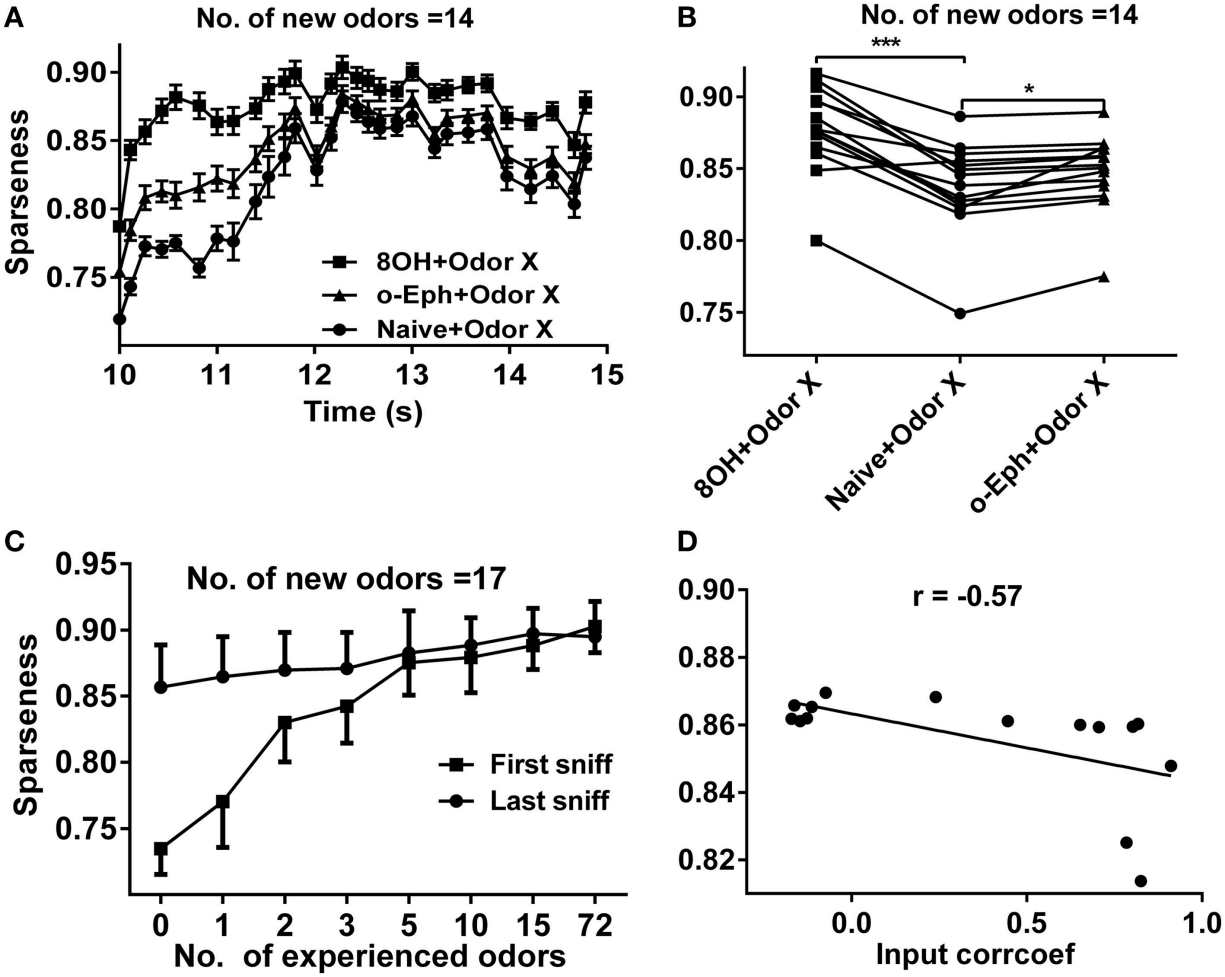
**Sparseness of the mitral cell network in different odor input conditions. (A)** A time course plot shows the sparseness of the mitral cell network in response to 14 new odor inputs in the single odor input (8OH or o-Eph) experience or naive condition. **(B)** A dot aligned plot shows the sparseness of the mitral cell network response at the last sniff shown in (A). ^***^p < 0.001, ^*^p < 0.05; one-way ANOVA with Tukey's *post-hoc* comparison test. **(C)** Sparseness measured in the first and last sniff period of the mitral cell network response to 17 new odor inputs in additional odor inputs experience conditions. The experienced odor inputs were delivered in sequence in ascending order of input strength. **(D)** The correlation between sparseness to 8OH under 14 different single odor input experience conditions vs. the correlation coefficients of input strength of these 14 experienced odor inputs and 8OH. The solid line represents the linear fitting curve.

Experimental and computational studies have shown that the response of the mitral cell network to odor inputs tends to be heterogeneous and spatiotemporally sparse (Yu et al., 2013, 2014). Our previous reports have shown that a sparse spatial spiking representation of specific odor signals can emerge naturally within several seconds of the training period from mitral-granule cell interactions and that the network response reaches a stable level of sparseness (Yu et al., 2013, 2014). To address how prior odor experience affects the evolution of sparseness in the mitral cell network and the final sparseness level in response to new odor inputs, we fixed the prior odor inputs to 8OH or o-Eph and then varied the new odor inputs or trained the network only with the new odor inputs (Figures 1B,C). Same as in our previous reports, the sparseness of the mitral cell network response gradually evolved from a relatively low sparseness level to a high sparseness level (Figures 1, 2A). We found that the sparseness of the mitral cell network response to new odor inputs in the single odor experience (8OH or o-Eph) condition was larger than that in the naïve condition at all sniff points the input were given (Figure 2A, *n* = 14 for the number of second odors). Figure 2B shows that the stable sparseness levels of the mitral cell network (represented by the last sniff point of 14.8 s) in both 8OH and o-Eph experience conditions are statistically larger than those in the naïve condition (one way ANOVA analysis, *p* < 0.01, Figure 2B). To demonstrate this phenomenon in a more systematic way, we trained the network with additional prior odor series in a manner similar to the single odor experience condition. As shown in Figure 2C, the stable sparseness level (represented by the last sniff point) of the mitral cell network increases with the number of prior odors experienced (one way ANOVA, *p* < 0.01). This scenario was more significant, as shown by the sparseness at the first sniff (Figure 2C). As shown in Supplementary Figure 2, the prior odors were delivered from low input strength level to high strength level in the 72 odor experience condition [72 Odors (lh)]. We also reversed the training sequence [i.e., prior odors were delivered from high input strength level to low strength level, 72 Odors (hl)]. Interestingly, the final sparseness of the mitral cell network is significantly lower in the 72 odor (hl) condition than that in the 72 odor (lh) condition (paired *t*-test, *p* < 0.001, Supplementary Figure 2). We plan to address this phenomenon extensively in future work, but the present work will mainly focus on the former training sequence (i.e., all prior odors were delivered from low input strength level to high strength level). Moreover, we fixed the new odor to 8OH and varied the experienced odor inputs. We found that the final sparseness level of the mitral cell network to 8OH was negatively correlated to the correlation coefficients of input strength of experienced odors and 8OH (Figure 2D). Similar results were found in the cases of k7-1 and o-Eph as the second odors (Supplementary Figure 3A).

It is worthwhile to note that, the new odors we used in our model were different from the experienced odors. We also tested the case that the second odor was the same as the first odor (experienced odor) in the single experienced odor condition and found no significant difference (Supplementary Figure 4).

A previous experimental study showed that prior odor experience could increase the tuning specificity of mitral cell to a variety of odors (Fletcher and Wilson, 2003). In our model, prior odor training could decrease the response of mitral cell to weak odor input leading to a slight increase of the tuning specificity of the mitral cell (Supplementary Figure 5A). To test whether the network sparseness change observed above was due to the increase of tuning specificity of mitral cells, we arbitrarily set the responses of mitral cell receiving no input from a given new odor the same as that in the naïve condition and left the rest responses of mitral cells (receiving at least one intensity from new odor) unchanged as in Figure 2C; we found that the stable sparseness levels hardly changed under different conditions (one way ANOVA, Supplementary Figure 5B). Such results suggest that the observed sparseness change of mitral cell network under prior odor experience condition is mainly due to the increase of the sparseness of mitral cells with no input from the new odors. And the experimentally observed tuning specificity of MCs after the odor exposure (e.g., Fletcher and Wilson, 2003) may have additional mechanisms that are beyond the present model simulation study.

**Figure 3:**
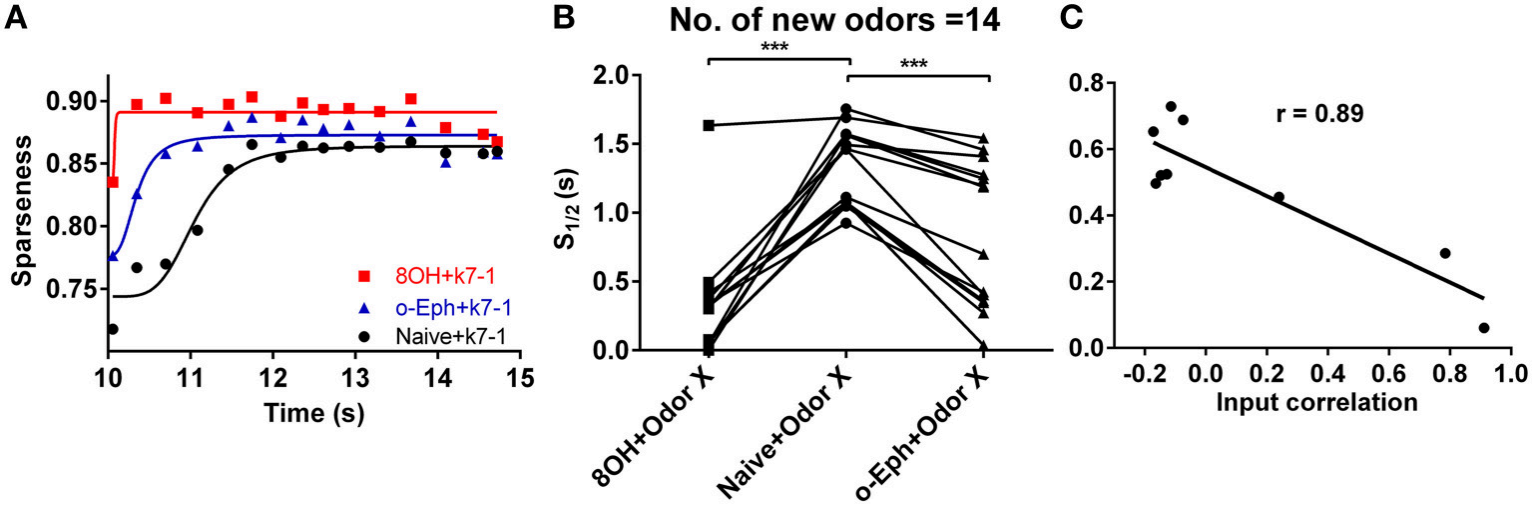
**Evolution of sparseness of mitral cell network responses in naïve and experienced odor input conditions. (A)** A time course plot shows the sparseness of the mitral cell network response to odor input K7-1 in the single odor input (8OH or o-Eph) experience or naive condition. The solid line represents the fitting curve described by the classical logarithmic function (see Materials and Methods). **(B)** 1/2 time of sparseness (S_1/2_) of the mitral cell network response to 17 new odor inputs in the single odor input (8OH or o-Eph) experience or naive conditions. S_1/2_ is the time elapsed from the first presentation of new odor inputs to the time when the sparseness reaches half of the maximum value. ^***^p < 0.001, one-way ANOVA with Tukey's *post-hoc* comparison test. **(C)** S_1/2_ of mitral cell network responses to 8OH in nine different single odor input experience conditions vs. the correlation coefficients of input strength of these nine experienced odor inputs and 8OH. The solid line represents the linear fitting curve.

To quantify how prior odor experience affects the evolution of the sparseness of the mitral cell network response to new odors, we fitted the time course of sparseness using a classical logarithmic function (Figure 3A). Then, based on the fitting curve, we determined the time at which the sparseness reaches half of the maximum value (denoted S_1/2_). We found that S_1/2_ of the network response to new odor inputs in both the 8OH and o-Eph experience conditions is less than in the naïve condition (one way ANOVA, *p* < 0.001, Figure 3B), implying that prior odor experience could accelerate the formation of sparse state sin the mitral cell network in response to new odor inputs. Moreover, to determine how the correlation of experience and new odor input strength affects the evolution of sparseness induced by new odor inputs, we fixed the new odor as 8OH and varied the experienced odor inputs, then measured the S_1/2_ of the network response to 8OH. Interestingly, we found that S_1/2_ was negatively proportional to the correlation coefficient of the input strength of the experienced odors and 8OH (*r* = 0.89, Figure 3C). Similar results were found in the cases of k7-1 and o-Eph as the second odors (Supplementary Figure 3B). This may imply that the network response to new odor input requires less time to evolve to a stable sparseness state following experienced odor input more similar to the new odor.

In summary, prior odor experience could accelerate the evolution of sparseness in the mitral cell network response to new odor inputs and increases the sparseness level.

### Prior odor input experience increases the response of the granule cell network

**Figure 4:**
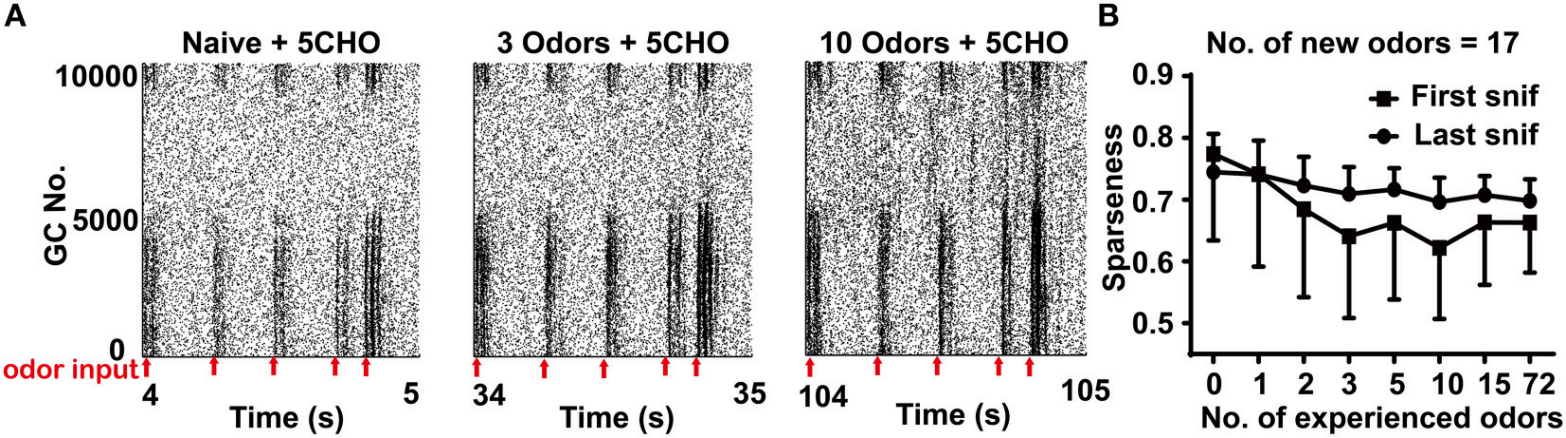
**Sparseness of the granule cell network in different odor input conditions. (A)** A raster plot shows the firing of the granule cell network in response to 5CHO in the 3 (middle) or 10 (right) odors experience or naïve conditions (left) in the stable state. 5CHO: pentanal. Red arrows show the sniff points. **(B)** The sparseness measured after the first and last sniff of the granule cell network response to 17 new odor inputs in a series of prior odor inputs conditions. The series of experienced odor inputs were delivered in sequence according to ascending order of input strength.

Previous experiments have shown that prior odor experience has a profound effect on the activity of the granule cell network in response to new odor inputs (Mandairon et al., 2008; Kato et al., 2012). We next tested the activity of the granule cell network in our model system. We also applied the sparseness measurement for mitral cells to quantify the activity in the granule cell network. Contrary to the sparseness in mitral cell network, the sparseness of the granule cell network decreased with the number of prior odors, implying that more prior odors leads to a larger increase of the response of the granule cell network to new odor inputs (Figure 4, one way ANOVA, *p* < 0.01).

### Effects of prior odor experience on synaptic weight in the mitral cell network

**Figure 5:**
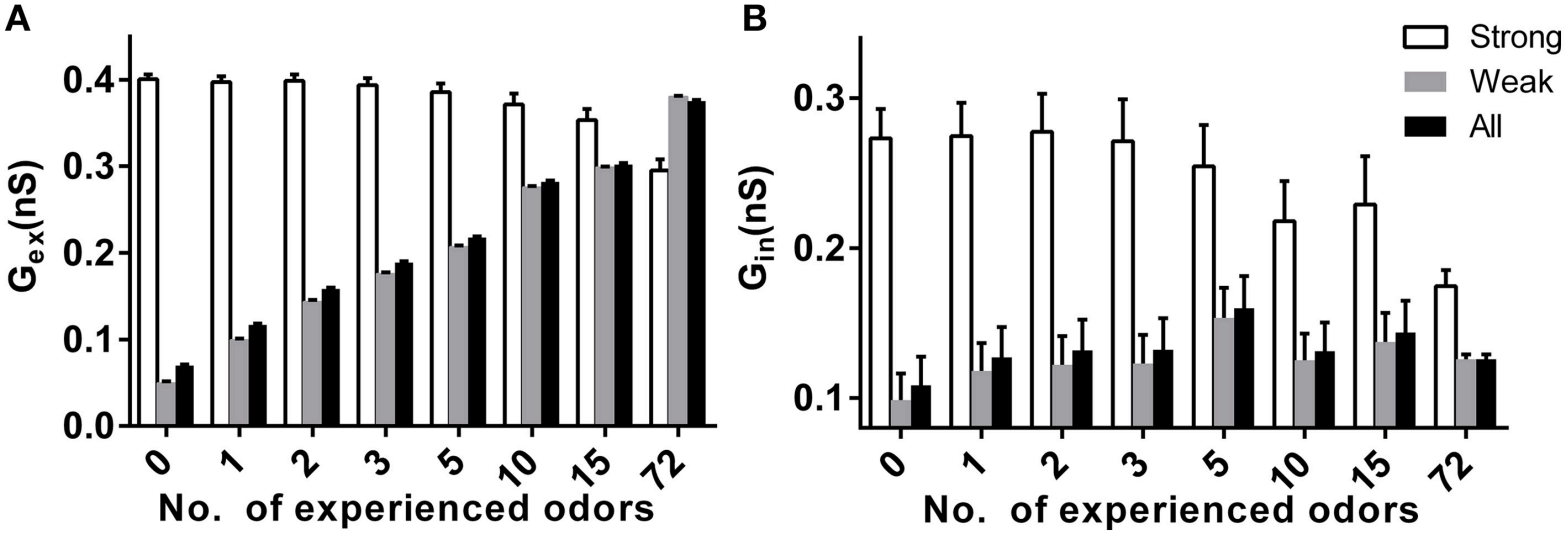
**Effects of prior odor experience on synapticweightin the mitral cell network. (A)** The average excitatory synaptic weight for mitral cells receiving strong (strength 3 or 4), weak (strength 0, 1, or 3), or all inputs in a variety of prior odor experience or naïve conditions. **(B)** Same as in **(A)**, but for the inhibitory synaptic weight.

Because synaptic plasticity exists in our model, the different stable sparseness of mitral or granule cell networks under different conditions may be due to the final synaptic weights in the bulb network. We tested the excitatory and inhibitory synaptic weight under different conditions in response to specific new odors. We divided the input strength into a strong group with strengths of 3 or 4 and a weak group with strengths of 0, 1, or 2. As shown in Figure 5A, the average excitatory synaptic weights for a mitral cell receiving weak or all inputs significantly increased with prior odor number, but decreased for mitral cells receiving strong inputs (two way ANOVA, *p* < 0.01). The same scenario applied to the average inhibitory synaptic weight (Figure 5B, two way ANOVA, *p* < 0.01).

Our previous studies reported that the change of sparseness of the mitral cell network resulted from the evolved dynamic changes in the synaptic weight of both excitatory and inhibitory dendrodendritic synapses (Yu et al., 2013, 2014). Such a developed dynamic change of synaptic weights has been suggested to affect the changes in the time course of mitral cell network sparseness (Yu et al., 2013, 2014). Now we would like to examine how the synaptic weights will further evolve with the continuous training of prior odor experience, and then examine how this prior experience could modulate the response sparseness of the mitral network to new odor inputs. We analyzed the time course of average excitatory weight (G_*ex*_) and inhibitory weight (G_*in*_) for mitral cells receiving strong inputs and weak inputs. Similar to our previous results (Yu et al., 2013, 2014), the time courses of response sparseness are tightly correlated with the changes of synaptic weight (especially excitatory synaptic weight) during the response to 8OH, both for naïve (Supplementary Figure 6) and single odor input experience (k7-1, Supplementary Figure 6). In the naïve condition, strong excitatory synaptic inputs gradually increased from 0.06 nS to a steady state of ~0.47 nS after 2 s of 8OH input (Supplementary Figures 6A,B). However, in the k7-1 odor experience condition, the sparseness of the mitral cell network reached a maximum level immediately in response to new 8OH input, and the strong excitatory and inhibitory synaptic inputs also reached a maximum value at the beginning of the 8OH input period (Supplementary Figures 6A,B). Therefore, these results imply that prior odor experience accelerates the evolution of synaptic weight in the mitral cell network to the steady state, which in turn accelerates the evolution of sparseness in the mitral cell network in response to new odor inputs.

### Prior odor input experience decreased the correlation of the mitral cell firing pattern

**Figure 6:**
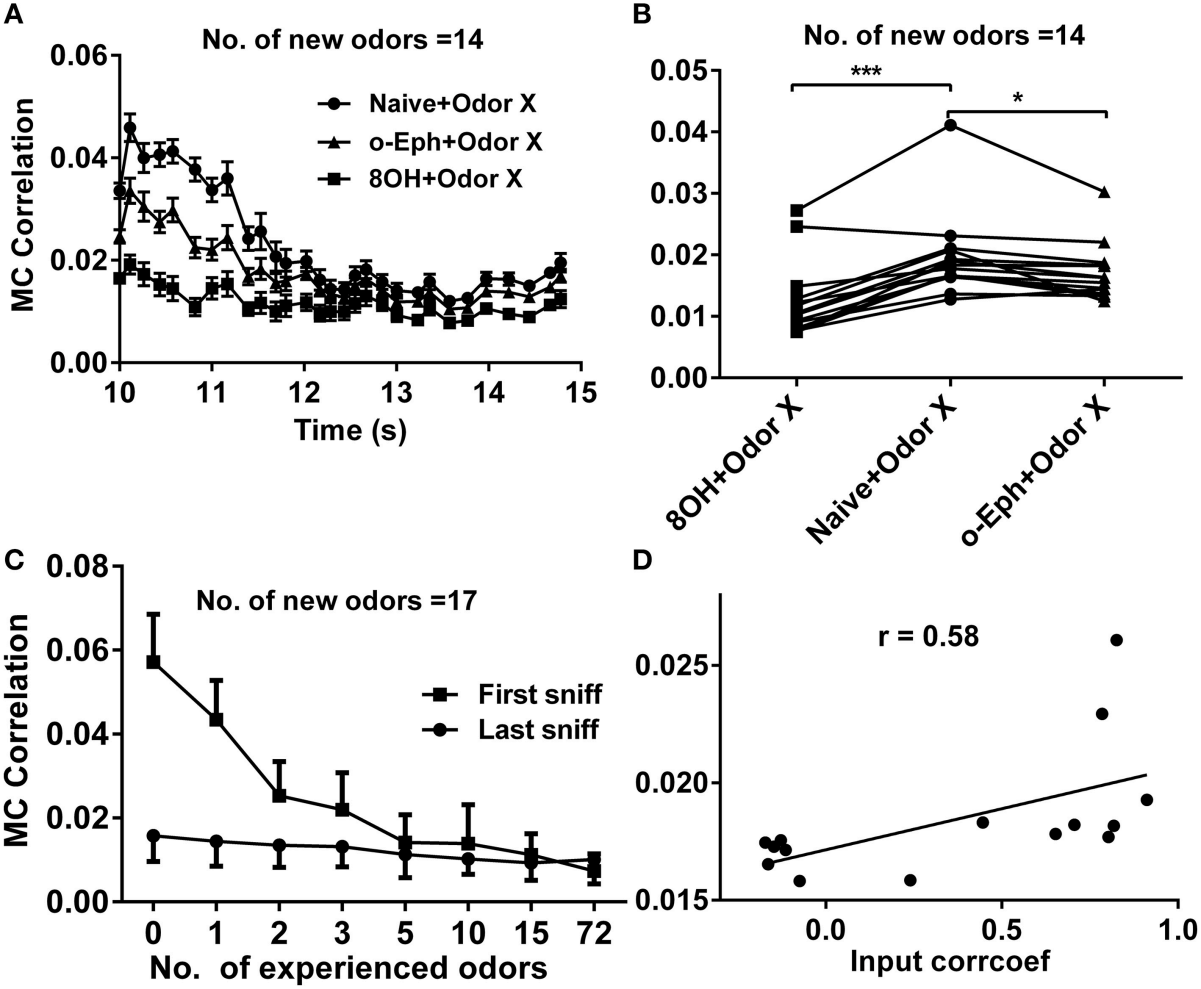
**Correlation between mitral cell firing patterns. (A)** A time course of the correlations between mitral cell firing patterns in response to 14 new odor inputs in the single odor input (8OH or o-Eph) experience or naïve condition. **(B)** A dot aligned plot shows the correlation between mitral cell firing pattern in response to 14 new odor inputs at the last sniff shown in **(A)**.^***^p < 0.001, ^*^p < 0.05; one-way ANOVA with Tukey's *post-hoc* comparison test. **(C)** The correlation between mitral cell firing patterns in response to 17 new odor inputs in networks measured after the first and last sniff with increased number of prior experienced odors. The odor inputs were delivered sequentially in order of ascending input strength. **(D)** The correlation between mitral cell firing patterns in response to 8OH under 14 different single odor input experience conditions vs. the correlation coefficients of input strength of these 14 experienced odor inputs and 8OH. The solid line represents the linear fitting curve.

Sparse coding is an efficient scheme by which an individual neuron independently encodes different properties of the input (Olshausen and Field, 1996; Vinje and Gallant, 2000). This naturally leads us to predict that when the mitral cell network reaches a high sparseness level, the correlation level among responses of mitral cells in the network should reach a low level. In fact, we have verified this prediction in our previous work (Yu et al., 2014). We therefore tried to determine whether prior odor experience would also affect the evolution of the decorrelated state among mitral cells in the network to new coming odor inputs. We quantified this correlation by averaging the correlation coefficients of all possible pairs of 500 mitral cell responses at each sniff point during new odor delivery. Similar to the evolution of sparseness in the mitral cell network, the correlation among mitral cells gradually evolved from a relatively high level to a low level (Figure 6A). We found that the correlation of mitral cell firing in the network in response to new odor inputs in one odor experience (8OH or o-Eph) condition was lower than that in the naïve condition at all the sniff points tested (Figure 6A). Figure 6B shows the stable correlation level of mitral cell firing in the network (represented by the last sniff point) for both 8OH and o-Eph experience conditions were statistically lower than that in the naïve condition (paired *t*-test, *p* < 0.05, Figure 6B). We then tested the correlation in conditions with more prior odor inputs. As shown in Figure 6C, the correlations of mitral cell firing in the network in response to new odor inputs at first and last sniff following prior odor experience both decreased as the number of prior odors increased (one way ANOVA, *p* < 0.01). Moreover, we fixed the new odor to 8OH and varied the experienced odor inputs; we found that the correlation of mitral cell firing in network in response to the new odor 8OH at last sniff was weakly linearly correlated to the correlation coefficients of the input strength of experienced odors and 8OH (Figure 6D). Similar results were found in the cases of k7-1 and o-Eph as the second odors (Supplementary Figure 3C). This result implies that the mitral cell firing response tends to be more decorrelated if the input strength of new coming odor differs more greatly from that of the prior experienced odor.

### Prior odor input experience decrease correlation of mitral cell network response

**Figure 7:**
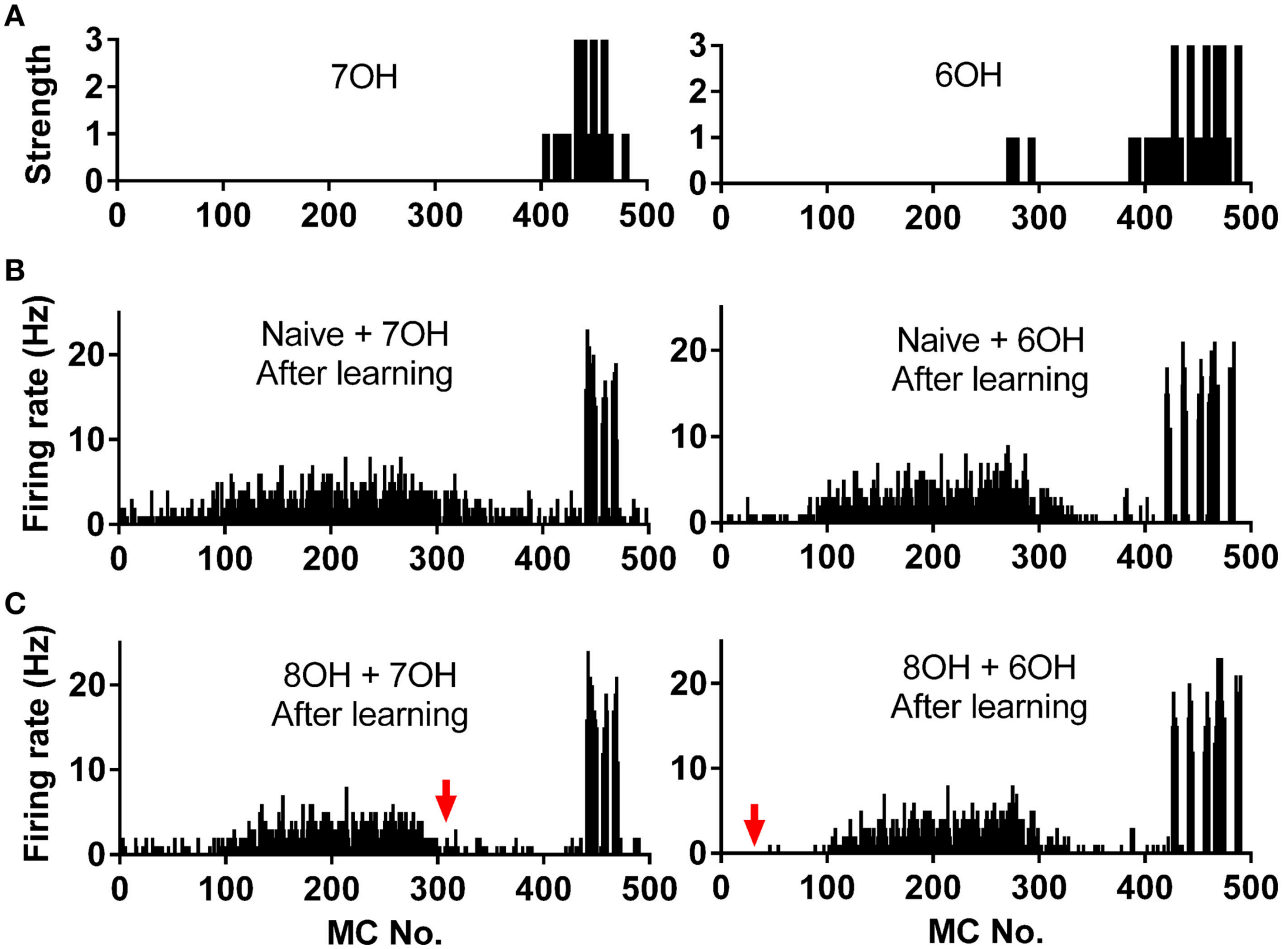
**Spatial firing rate patterns of 500 mitral cells. (A)** The strength of odor inputs 7OH (left) and 6OH (right) to each mitral cell. **(B)** The spatial firing pattern of 500 mitral cells during the 4th second after training in response to 7OH (left) or 6OH (right) in the naïve condition. **(C)** The spatial firing pattern of 500 mitral cells during the 4th second after training in response to 7OH (left) or 6OH (right) in the 8OH experience condition. The red arrow (right) indicates that the firing pattern of the network in response to 7OH in the 8OH experience condition (**C**, left) differs more than that of the naive condition (**B**, left) from the firing pattern of the network to 6OH in the naïve condition (**B**, right). Similarly, the red arrow (left) indicates that the firing pattern of the network in response to 6OH in the 8OH experience condition (**C**, right) differs more than that of the naive condition (**B**, right) from the firing pattern of the network to 7OH in the naïve conditions (**B**, left).

We already tested the effects of prior odor experience on mitral cell firing pattern in response to the corresponding new odor inputs. A more direct way to measure the coding efficiency of the mitral cell network in response to different odor inputs is to calculate the similarity of the network response to different odor inputs, especially to similar odor inputs (Yu et al., 2014). We therefore investigated how prior odor experience affects the mitral cell network response to different new odor inputs. Odors 7OH and 6OH are two very similar odor inputs (Figure 7A). The response of the mitral cell network to 6OH in one odor input (8OH) experience condition was more different than that in the naïve condition to the network response to 7OH in naïve condition after the training process (Figures 7B,C). For instance, the firing rates of mitral cells 1–100 to 6OH in the 8OH experience condition (Figure 7C, right) were more different than that in naïve condition (Figure 6B, right) from that to 7OH in naïve condition (Figure 7B, left). A similar result was also found when we compared the response of the mitral cell network to 7OH in the 8OH experience condition or the naïve condition with that to 6OH in the naïve condition (Figures 7B,C).

**Figure 8:**
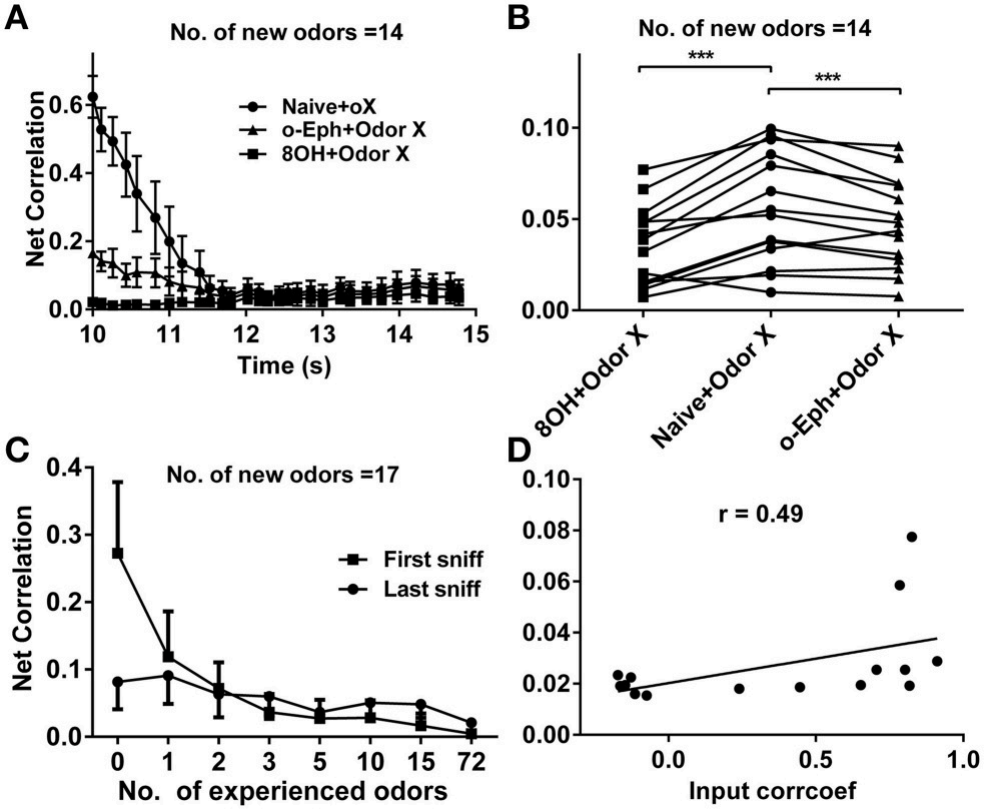
**Correlation between mitral cell network responses. (A)** Time courses of the correlations between mitral cell network responses to 14 new odor inputs after one odor input experience (8OH or o-Eph) or under naive conditions. **(B)** A dot aligned plot shows the correlation between mitral cell network responses to 17 new odor inputs at the last sniff shown in **(A)**. ^***^p < 0.001; one-way ANOVA with Tukey's *post-hoc* comparison test. **(C)** Correlation between mitral cell network responses to 17 new odor inputs measured after the first and last sniff after experiencing additional odor inputs. The odor inputs were delivered in order of ascending input strength. **(D)** Correlation between mitral cell network responses to 8OH in 14 different single odor input experience conditions vs. the correlation coefficients of input strength of these 14 experienced odor inputs and 8OH. The solid line represents the linear fitting curve.

To quantify the similarity of the mitral cell network response to different odor inputs in different conditions, we measured the network response correlation as described in the Materials and Methods section. The correlations of mitral cell network responses evolved gradually from relatively high to low level (Figure 8A). Furthermore, the correlations between mitral cell network responses to new coming odor inputs in the one odor experience (8OH or o-Eph) condition were lower than in the naïve condition at all sniff points tested (Figure 8A). Figure 8B shows the stable correlation level of the mitral cell network response (represented by the last sniff point) in both 8OH and o-Eph experience conditions were statistically lower than in the naïve condition (one way ANOVA, *p* < 0.05, Figure 8B). We then tested the correlation in conditions with more series of prior odor inputs. As shown in Figure 8C, the correlations of mitral cell network response to new coming odor inputs in prior odor experience at the first and last sniff point decreased while the number of prior odors increased (one way ANOVA, *p* < 0.01). Similar to Figure 6D, we fixed the new odor to 8OH, and varied the experienced odor inputs and we found that the correlation of mitral cell network response to new coming odor 8OH was weakly linearly correlated to correlation coefficients of the input strength of experienced odors and 8OH (Figure 8D). Similar results were also found in the cases of k7-1 and o-Eph as the second odors (Supplementary Figure 3D).

## Discussion

Using a scaled up mitral-granule cell network model and a set of odors with relatively strong input strength, we systematically investigated how prior odor input affects the coding paradigm of mitral cells to new incoming odor inputs. The following findings were observed: (1) when increasing the number of prior odors, the activity of the mitral cell network decreased and the granule cell network increased, gradually reaching an equilibrium level. (2) prior odor experience accelerated the formation of a stable sparseness level of the mitral cell network to new odors; (3) increasing prior odor experience also facilitated the mitral cell network to evolve to a more decorrelated state; (4) prior odor experience decreased the correlation of the mitral cell network response to new odors and this effect is more obvious after training the network with a larger number of prior odors. Note that all the changes gradually reach an equilibrium level that does not change with additional odor experience. All changes may be attributed to two key factors: (1) the continuous LTP effect for those mitral cells receiving a sustained strong input; (2) more and more mitral cells are activated when more odors are presented to the network, inducing more dynamic changes in the excitatory and inhibitory synaptic weights of the dendrodendritic synapses. An equilibrium is reached when most of the relevant mitral cells have been activated during the past odor experiences.

### Sparseness of mitral and granule cell in prior odor experience conditions

Sparse coding has been suggested as an efficient way to code the sensory inputs (Olshausen and Field, 1996; Rinberg et al., 2006; Davison and Katz, 2007; Koulakov and Rinberg, 2011). Previous studies have found that the response of mitral cell network to new coming odor inputs decreases after prior odor experience (Buonviso et al., 1998; Buonviso and Chaput, 2000; Fletcher and Wilson, 2003; Kato et al., 2012). We systematically tested several prior odors inputs in our large scale olfactory bulb model, and we found the sparseness of the mitral cell network to new coming odors increases with the number of prior odors (Figure 2C). Moreover, we found the sparseness of the granule cell network to new coming odors decrease with the number of prior odors (Figure 4B). In fact, previous experimental results also found that prior odor experience could increase the activity of interneuron (Mandairon et al., 2008). We further found the average of excitatory and inhibitory synaptic weight both increase along with the number of prior odor inputs (Figure 5). As the mitral cell is the main target of granule cell and granule cell is also the main target of mitral cell, we can infer that the increase of excitatory synaptic weight leads to the increase of granule cell activity, and the increase of inhibitory synaptic weight combined with the increase of granule cell activity leads to the decrease of the mitral cell activity.

Previous experimental study also showed that prior odor experience could decrease the granule cell activity (Kato et al., 2012). In our simulations, prior odor experience decreases the response of mitral cell network, which tends to decrease the activity of granule cells. By contrast, prior odor experience increases the average excitatory synaptic weight to granule cell, which tends to increase the activity of granule cells. These two contrary effects of prior odor experience on granule cell activity might be the cause for the varied results of granule cell activity change to prior odor experience observed in experiments.

It should be noted that an experimental study shows that mitral cell responses decreases more after the same prior odor exposure than with different odor experience (Kato et al., 2012). In our work, the response of mitral cells hardly changed when it was in the same prior odor conditions. This suggests the phenomenon observed by Kato et al. (2012) might be attributed to a different mechanism that is not involved in the current model network.

Previous experimental results have also shown that the first sniff after odor input is very important for odor discrimination behavior (Uchida and Mainen, 2003; Cury and Uchida, 2010). In addition to analyzing olfactory bulb responses at equilibrium, we also analyzed the response properties right after the first sniff in all conditions. We found the sparseness change (or correlation of mitral cell network response change) induced by prior odor experience is more significant for first sniff cycle than that for the last sniff cycle. The more experienced odors result in less response difference in the sparseness level (or response correlation) between the first and last sniff cycles. This may suggest that the response in the first sniff may contain important information for odor discrimination that can be enhanced by the experienced odors.

It is worthwhile to note that the increasing rate of sparseness of mitral cell network tend to decrease when the number of prior odors increases (Figure 2C). For instance, the increase of sparseness between three and five odors conditions (increase of only two odors) is larger than that between 15 and 72 odors conditions (increase of 57 odors). We may infer that the sparseness level of the mitral network would saturate after a certain number of prior odor experience. Another interesting phenomenon needed to further test is that the different training sequence of series of prior odors would have significantly different effect on the response of mitral cell network to new coming odors.

### Sparseness evolution in mitral cell network

Previous studies have shown a sparse spiking representation of specific odor can emerge naturally after several seconds of a learning period (with certain odor input frequency) from the mitral-granule cell synaptic connections (Yu et al., 2013, 2014). And this phenomenon may be corresponding to the learning process of animal to a new odor inputs. In the one odor experience condition, we found the prior odor experience accelerates the process to reach the stable sparse state (Figure 3). Furthermore, we found that such acceleration of the sparseness to reach stable sparse state was well correlated to the acceleration of the excitatory and inhibitory synaptic weight to reach the maximum value (Supplementary Figure 6). And further experiments are needed to confirm such findings.

We also found that the more similar the new odor was to the experienced odor, the faster the mitral cell network reached a stable sparseness level, which may be due to less time needed to train more overlapping synaptic interactions to reach steady state. On the other hand, more disparate prior experienced odors lead to a higher stable sparseness level of mitral cell to the new odor, which may be due to the increase of the overall inhibitory synaptic weight resulting from the activation of more mitral and granule cells by more experienced odors. We may infer that the rate of formation of stable sparseness and the sparseness level itself are two different aspects of the odor representation of the mitral cell network.

### Decorrelation of the mitral cell network response

Our previous study has shown that the response of the mitral cell network tends to be decorrelated and accompanied by sparseness (Yu et al., 2014). Our current work shows correlations within the mitral cell network to new odors decrease with the number of prior odors (Figure 6C). A similar correlation exists for mitral cell network responses to different odor inputs—a direct way to quantify coding efficiency under different conditions (Figure 8C). This may partially give an explanation to why enrichment could increase the ability of an animal to discriminate different odors (Mandairon et al., 2006a,b,c; Sinding et al., 2011).

Previous experimental and computational results have extensively shown the importance of granule cell activity and inhibitory synaptic weight for representation of odor inputs in olfactory bulb network (Mandairon et al., 2006a, 2008; Koulakov and Rinberg, 2011; Kato et al., 2012). We infer that such a decorrelated state between mitral cell firing in a specific network and the network response to different odor inputs is due to an increase in granule cell activity and inhibitory synaptic weight after odor experience.

In summary, using a scaled up olfactory bulb model, we systematically investigated how prior odor experience affects the sparse representation of new odor inputs by the olfactory bulb network. In conclusion, the gradual increased inhibitory weight of granule cells together with the slightly increased firing rates of gradual cell populations promote the response sparseness and decorrelated state of mitral populations to new odor inputs. These results may help to better explain how prior sensory experience affects the behavior of animals in response to new odor inputs.

## Author contributions

YY and ZS designed research; ZS and MM performed research; ZS and YY wrote the paper. All authors reviewed the manuscript.

### Conflict of interest statement

The authors declare that the research was conducted in the absence of any commercial or financial relationships that could be construed as a potential conflict of interest.

## References

[B1] Arenkiel B. R. Hasegawa H. Yi J. J. Larsen R. S. Wallace M. L. Philpot B. D.. (2011). Activity-induced remodeling of olfactory bulb microcircuits revealed by monosynaptic tracing. PLoS ONE 6:e29423. 10.1371/journal.pone.0029423 22216277 PMC3247270

[B2] Bischofberger J. Jonas P. (1997). Action potential propagation into the presynaptic dendrites of rat mitral cells. J. Physiol. 504, 359–365. 10.1111/j.1469-7793.1997.359be.x 9365910 PMC1159916

[B3] Buonviso N. Chaput M. (2000). Olfactory experience decreases responsiveness of the olfactory bulb in the adult rat. Neuroscience 95, 325–332. 10.1016/S0306-4522(99)00450-9 10658611

[B4] Buonviso N. Gervais R. Chalansonnet M. Chaput M. (1998). Short-lasting exposure to one odour decreases general reactivity in the olfactory bulb of adult rats. Eur. J. Neurosci. 10, 2472–2475. 10.1046/j.1460-9568.1998.00266.x 9749774

[B5] Cury K. M. Uchida N. (2010). Robust odor coding via inhalation-coupled transient activity in the mammalian olfactory bulb. Neuron 68, 570–585. 10.1016/j.neuron.2010.09.040 21040855

[B6] Davison I. G. Katz L. C. (2007). Sparse and selective odor coding by mitral/tufted neurons in the main olfactory bulb. J. Neurosci. 27, 2091–2101. 10.1523/JNEUROSCI.3779-06.2007 17314304 PMC6673545

[B7] Ennis M. Linster C. Aroniadou-Anderjaska V. Ciombor K. Shipley M. T. (1998). Glutamate and synaptic plasticity at mammalian primary olfactory synapses. Ann. N.Y. Acad. Sci. 855, 457–466. 9929639 10.1111/j.1749-6632.1998.tb10606.x

[B8] Fletcher M. L. Wilson D. A. (2003). Olfactory bulb mitral-tufted cell plasticity: odorant-specific tuning reflects previous odorant exposure. J. Neurosci. 23, 6946–6955. 12890789 10.1523/JNEUROSCI.23-17-06946.2003 PMC2367229

[B9] Franco L. Rolls E. T. Aggelopoulos N. C. Jerez J. M. (2007). Neuronal selectivity, population sparseness, and ergodicity in the inferior temporal visual cortex. Biol. Cybern. 96, 547–560. 10.1007/s00422-007-0149-1 17410377

[B10] Gao Y. Strowbridge B. W. (2009). Long-term plasticity of excitatory inputs to granule cells in the rat olfactory bulb. Nat. Neurosci. 12, 731–733. 10.1038/nn.2319 19412165 PMC2693249

[B11] Haykin S. (1994). Neural Networks: A Comprehensive Foundation. New York, NY: Macmillan Publishing.

[B12] Hines M. L. Carnevale N. T. (1997). The NEURON simulation environment. Neural Comput. 9, 1179–1209. 10.1162/neco.1997.9.6.1179 9248061

[B13] Hines M. L. Carnevale N. T. (2001). NEURON: a tool for neuroscientists. Neuroscientist 7, 123–135. 10.1177/107385840100700207 11496923

[B14] Kato H. K. Chu M. W. Isaacson J. S. Komiyama T. (2012). Dynamic sensory representations in the olfactory bulb: modulation by wakefulness and experience. Neuron 76, 962–975. 10.1016/j.neuron.2012.09.037 23217744 PMC3523713

[B15] Kay L. M. Sherman S. M. (2007). An argument for an olfactory thalamus. Trends Neurosci. 30, 47–53. 10.1016/j.tins.2006.11.007 17161473

[B16] Khan A. G. Parthasarathy K. Bhalla U. S. (2010). Odor representations in the mammalian olfactory bulb. Wiley Interdiscip. Rev. Syst. Biol. Med. 2, 603–611. 10.1002/wsbm.85 20836051

[B17] Koulakov A. A. Rinberg D. (2011). Sparse incomplete representations: a potential role of olfactory granule cells. Neuron 72, 124–136. 10.1016/j.neuron.2011.07.031 21982374 PMC3202217

[B18] Ma T. F. Zhao X. L. Cai L. Zhang N. Ren S. Q. Ji F.. (2012). Regulation of spike timing-dependent plasticity of olfactory inputs in mitral cells in the rat olfactory bulb. PLoS ONE 7:e35001. 10.1371/journal.pone.0035001 22536347 PMC3334975

[B19] Mandairon N. Didier A. Linster C. (2008). Odor enrichment increases interneurons responsiveness in spatially defined regions of the olfactory bulb correlated with perception. Neurobiol. Learn. Mem. 90, 178–184. 10.1016/j.nlm.2008.02.008 18406178

[B20] Mandairon N. Linster C. (2009). Odor perception and olfactory bulb plasticity in adult mammals. J. Neurophysiol. 101, 2204–2209. 10.1152/jn.00076.2009 19261715

[B21] Mandairon N. Stack C. Kiselycznyk C. Linster C. (2006a). Broad activation of the olfactory bulb produces long-lasting changes in odor perception. Proc. Natl. Acad. Sci. U.S.A. 103, 13543–13548. 10.1073/pnas.0602750103 16938883 PMC1569199

[B22] Mandairon N. Stack C. Kiselycznyk C. Linster C. (2006b). Enrichment to odors improves olfactory discrimination in adult rats. Behav. Neurosci. 120, 173–179. 10.1037/0735-7044.120.1.173 16492127

[B23] Mandairon N. Stack C. Linster C. (2006c). Olfactory enrichment improves the recognition of individual components in mixtures. Physiol. Behav. 89, 379–384. 10.1016/j.physbeh.2006.07.013 16904713

[B24] Migliore M. Cavarretta F. Hines M. L. Shepherd G. M. (2014). Distributed organization of a brain microcircuit analyzed by three-dimensional modeling: the olfactory bulb. Front. Comput. Neurosci. 8:50. 10.3389/fncom.2014.00050 24808855 PMC4010739

[B25] Migliore M. Cavarretta F. Marasco A. Tulumello E. Hines M. L. Shepherd G. M. (2015). Synaptic clusters function as odor operators in the olfactory bulb. Proc. Natl. Acad. Sci. U.S.A. 112, 8499–8504. 10.1073/pnas.1502513112 26100895 PMC4500266

[B26] Migliore M. Hines M. L. Mctavish T. S. Shepherd G. M. (2010). Functional roles of distributed synaptic clusters in the mitral-granule cell network of the olfactory bulb. Front. Integr. Neurosci. 4:122. 10.3389/fnint.2010.00122 21258619 PMC3024007

[B27] Migliore M. Hines M. L. Shepherd G. M. (2005). The role of distal dendritic gap junctions in synchronization of mitral cell axonal output. J. Comput. Neurosci. 18, 151–161. 10.1007/s10827-005-6556-1 15714267

[B28] Migliore M. Hoffman D. A. Magee J. C. Johnston D. (1999). Role of an A-type K+ conductance in the back-propagation of action potentials in the dendrites of hippocampal pyramidal neurons. J. Comput. Neurosci. 7, 5–15. 10.1023/A:1008906225285 10481998

[B29] Migliore M. Inzirillo C. Shepherd G. M. (2007). Learning mechanism for column formation in the olfactory bulb. Front. Integr. Neurosci. 1:12. 10.3389/neuro.07.012.2007 18958240 PMC2526006

[B30] Migliore M. Shepherd G. M. (2008). Dendritic action potentials connect distributed dendrodendritic microcircuits. J. Comput. Neurosci. 24, 207–221. 10.1007/s10827-007-0051-9 17674173 PMC3752904

[B31] Mori K. Nowycky M. C. Shepherd G. M. (1981). Analysis of a long-duration inhibitory potential in mitral cells in the isolated turtle olfactory bulb. J. Physiol. 314, 311–320. 10.1113/jphysiol.1981.sp013709 7310694 PMC1249435

[B32] Mori K. Takahashi Y. K. Igarashi K. M. Yamaguchi M. (2006). Maps of odorant molecular features in the Mammalian olfactory bulb. Physiol. Rev. 86, 409–433. 10.1152/physrev.00021.2005 16601265

[B33] Olshausen B. A. Field D. J. (1996). Emergence of simple-cell receptive field properties by learning a sparse code for natural images. Nature 381, 607–609. 10.1038/381607a0 8637596

[B34] Patneau D. K. Stripling J. S. (1992). Functional correlates of selective long-term potentiation in the olfactory cortex and olfactory-bulb. Brain Res. 585, 219–228. 10.1016/0006-8993(92)91210-6 1511305

[B35] Pinato G. Midtgaard J. (2005). Dendritic sodium spikelets and low-threshold calcium spikes in turtle olfactory bulb granule cells. J. Neurophysiol. 93, 1285–1294. 10.1152/jn.00807.2004 15483062

[B36] Rinberg D. Koulakov A. Gelperin A. (2006). Sparse odor coding in awake behaving mice. J. Neurosci. 26, 8857–8865. 10.1523/JNEUROSCI.0884-06.2006 16928875 PMC6674368

[B37] Schoppa N. E. Westbrook G. L. (1999). Regulation of synaptic timing in the olfactory bulb by an A-type potassium current. Nat. Neurosci. 2, 1106–1113. 10.1038/16033 10570488

[B38] Shusterman R. Smear M. C. Koulakov A. A. Rinberg D. (2011). Precise olfactory responses tile the sniff cycle. Nat. Neurosci. 14, 1039–1044. 10.1038/nn.2877 21765422 PMC13348895

[B39] Sinding C. Thomas-Danguin T. Crepeaux G. Schaal B. Coureaud G. (2011). Experience influences elemental and configural perception of certain binary odour mixtures in newborn rabbits. J. Exp. Biol. 214, 4171–4178. 10.1242/jeb.063610 22116759

[B40] Stanton P. K. (1996). LTD, LTP, and the sliding threshold for long-term synaptic plasticity. Hippocampus 6, 35–42. 8878740 10.1002/(SICI)1098-1063(1996)6:1<35::AID-HIPO7>3.0.CO;2-6

[B41] Uchida N. Mainen Z. F. (2003). Speed and accuracy of olfactory discrimination in the rat. Nat. Neurosci. 6, 1224–1229. 10.1038/nn1142 14566341

[B42] Vinje W. E. Gallant J. L. (2000). Sparse coding and decorrelation in primary visual cortex during natural vision. Science 287, 1273–1276. 10.1126/science.287.5456.1273 10678835

[B43] Wang X. J. Buzsaki G. (1996). Gamma oscillation by synaptic inhibition in a hippocampal interneuronal network model. J. Neurosci. 16, 6402–6413. 8815919 10.1523/JNEUROSCI.16-20-06402.1996 PMC6578902

[B44] Wang X. Y. Mckenzie J. S. Kemm R. E. (1996). Whole-cell K+ currents in identified olfactory bulb output neurones of rats. J. Physiol. 490(Pt 1), 63–77. 10.1113/jphysiol.1996.sp021127 8745279 PMC1158648

[B45] Willhite D. C. Nguyen K. T. Masurkar A. V. Greer C. A. Shepherd G. M. Chen W. R. (2006). Viral tracing identifies distributed columnar organization in the olfactory bulb. Proc. Natl. Acad. Sci. U.S.A. 103, 12592–12597. 10.1073/pnas.0602032103 16895993 PMC1567923

[B46] Xiong W. H. Chen W. R. (2002). Dynamic gating of spike propagation in the mitral cell lateral dendrites. Neuron 34, 115–126. 10.1016/S0896-6273(02)00628-1 11931746

[B47] Yu Y. G. McTavish T. S. Hines M. L. Shepherd G. M. Valenti C. Migliore M. (2013). Sparse distributed representation of odors in a large-scale olfactory bulb circuit. PLoS Comput. Biol. 9:e1003014. 10.1371/journal.pcbi.1003014 23555237 PMC3610624

[B48] Yu Y. G. Migliore M. Hines M. L. Shepherd G. M. (2014). Sparse coding and lateral inhibition arising from balanced and unbalanced dendrodendritic excitation and inhibition. J. Neurosci. 34, 13701–13713. 10.1523/JNEUROSCI.1834-14.2014 25297097 PMC4188968

[B49] Zelles T. Boyd J. D. Hardy A. B. Delaney K. R. (2006). Branch-specific Ca2+ influx from Na+-dependent dendritic spikes in olfactory granule cells. J. Neurosci. 26, 30–40. 10.1523/JNEUROSCI.1419-05.2006 16399670 PMC6674300

