# Supplementary material for: Odor Experience Facilitates Sparse Representations of New Odors in a Large-Scale Olfactory Bulb Model
Source: Front Neuroanat. 2016 Feb 11;10:10. doi: 10.3389/fnana.2016.00010 (PMC4749983; doi:10.3389/fnana.2016.00010)
Supplement: Supplementary file 1 [file DataSheet1.pdf]

Supplementary Material

Odor experience facilitates the sparse coding of new odors in a large-scale olfactory bulb model

Shanglin Zhou, Michele Migliore, Yuguo Yu*

*** Correspondence:** Yuguo Yu: yuyuguo@fudan.edu.cn

1 Supplementary Figures

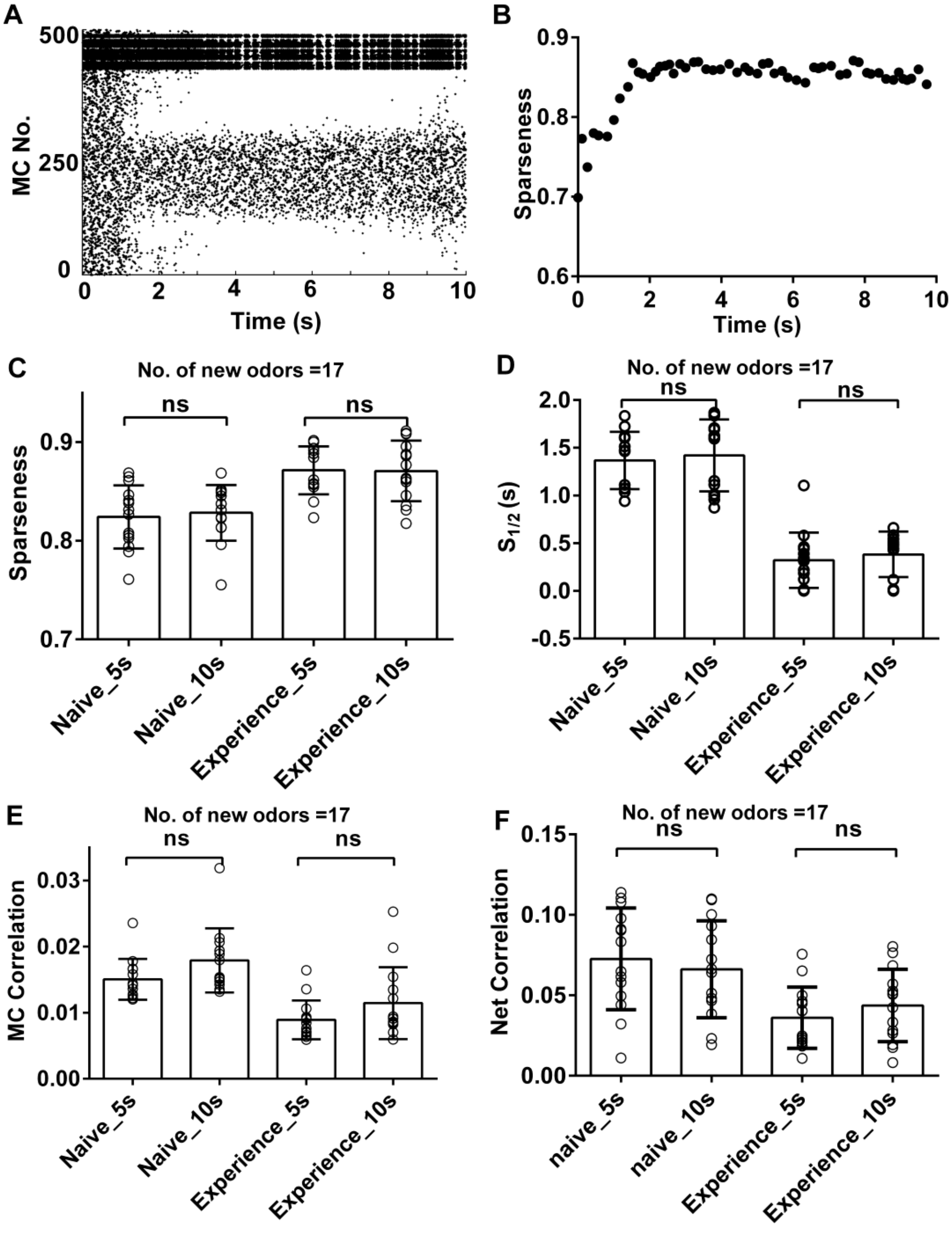

Supplementary Figure 1 Sparse representation of new odors in mitral cell network after 5 s and 10 s stimulus. (A) Raster plot shows the mitral cell network response during 10 s k7-1 stimuli. (B) Sparseness measurement of mitral cell network response to 17 new odors during 10 s k7-1 stimuli. Sparseness (C), $S_{1/2}$ (D), correlation between mitral cell firing pattern (E) and correlation

between mitral cell network responses (F) after 5 s or 10 s simulations in naïve or one odor (k7-1) experience condition. ns: non significance, one-way ANOVA with Tukey’s posthoc comparison test.

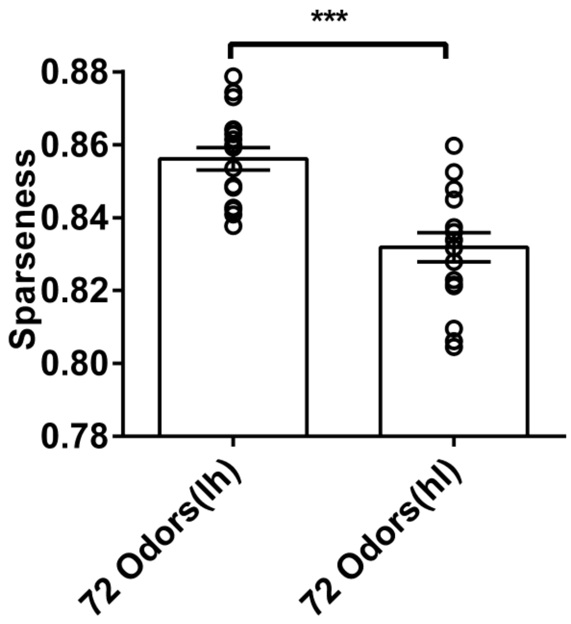

Supplementary Figure 2. **Sparseness of mitral cell network in 72 odors experience conditions**. Two paradigm of 72 odors experience condition were applied: odor input strength form low level to high level (72 Odors (lh)) or from high level to low level (72 Odors (hl)). Sparseness of mitral cell network was calculated as described in methods section. ***, $p < 0.01$ paired t-test.

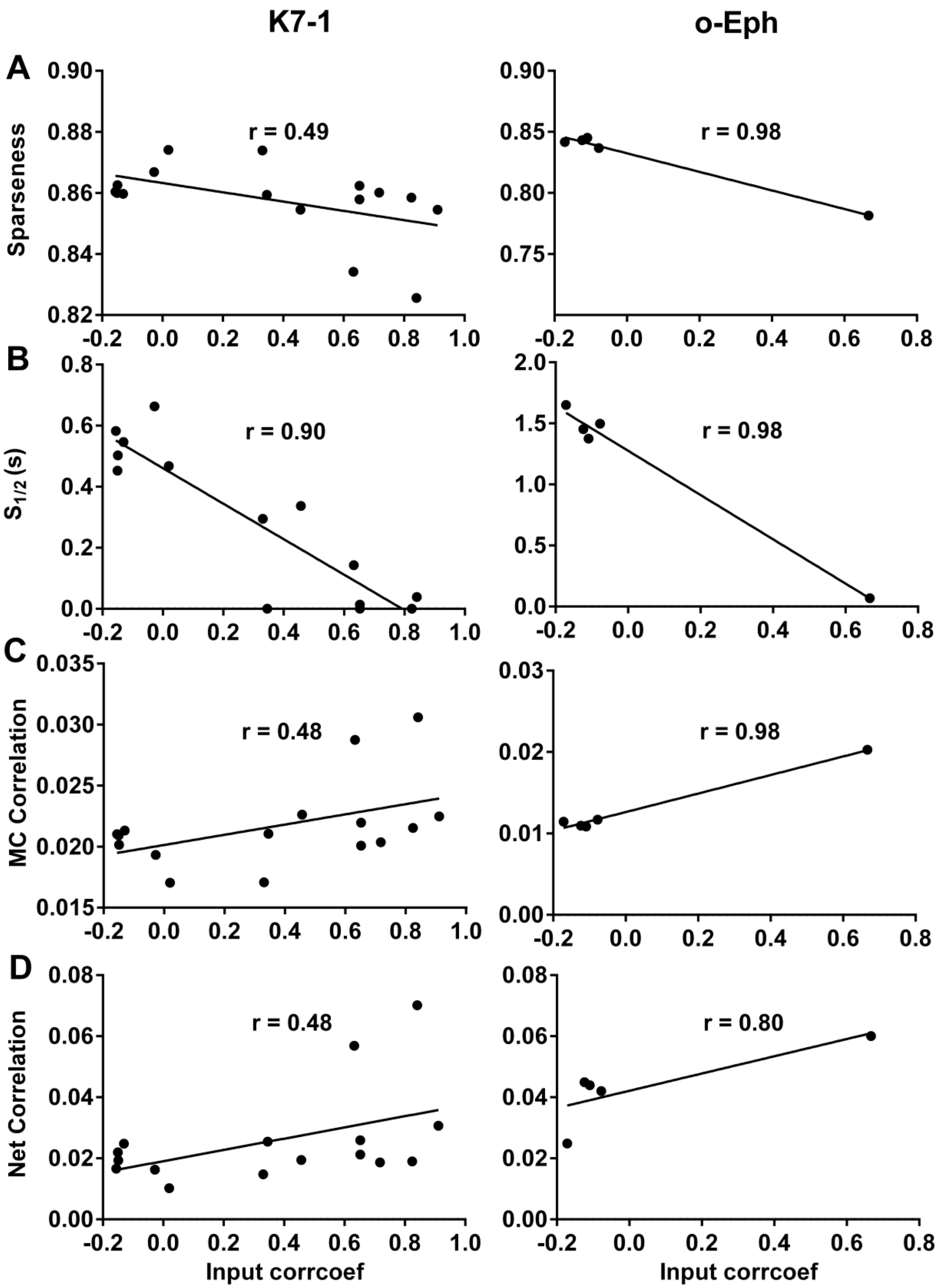

K7-1
o-Eph
A
Sparseness
r = 0.49
r = 0.98
B
S1/2 (s)
r = 0.90
r = 0.98
C
MC Correlation
r = 0.48
r = 0.98
D
Net Correlation
r = 0.48
r = 0.80
Input corrcoef
Input corrcoef

Supplementary Figure 3 Relation of mitral cell responses to new odors with prior odor similarity. Sparseness (A), $S_{1/2}$ (B), correlation between mitral cell firing pattern (C) and correlation between mitral cell network response (D) of mitral cell network responses to k7-1 (left) and o-Eph (right) in one odor input experience conditions versus the correlation coefficients of input strength of these experience odor inputs and k7-1 or o-Eph. The solid lines represent the linear fitting curves.

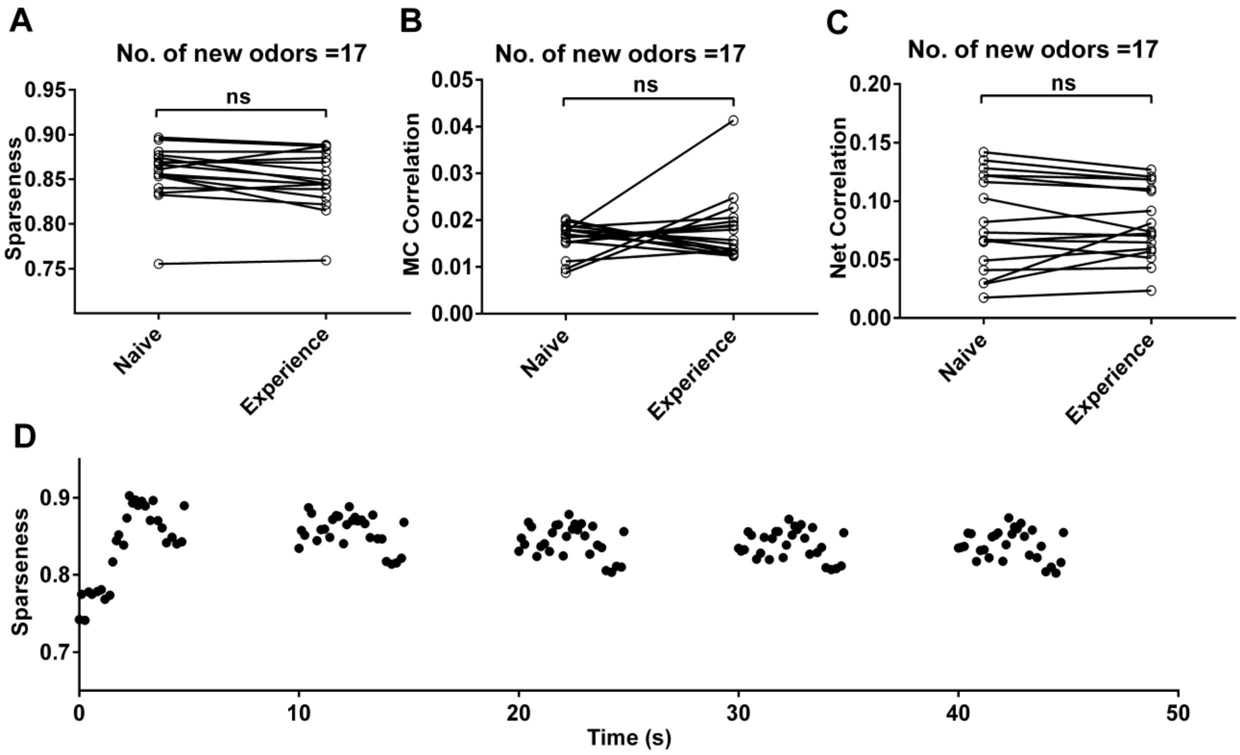

Supplementary Figure 4 Mitral cell responses to the same odor inputs in naïve and one odor experience conditions. Same 17 new odors were given for 5s respectively in one odor experience or naïve conditions. Sparseness (A), correlation between mitral cell firing pattern (B), and correlation between mitral cell network responses (C) were measured as described in Methods section. ns: non significance, paired t-test. (D) Sparseness time course of mitral cell network to 5 times of repetitive o-Eph inputs (5 seconds for each).

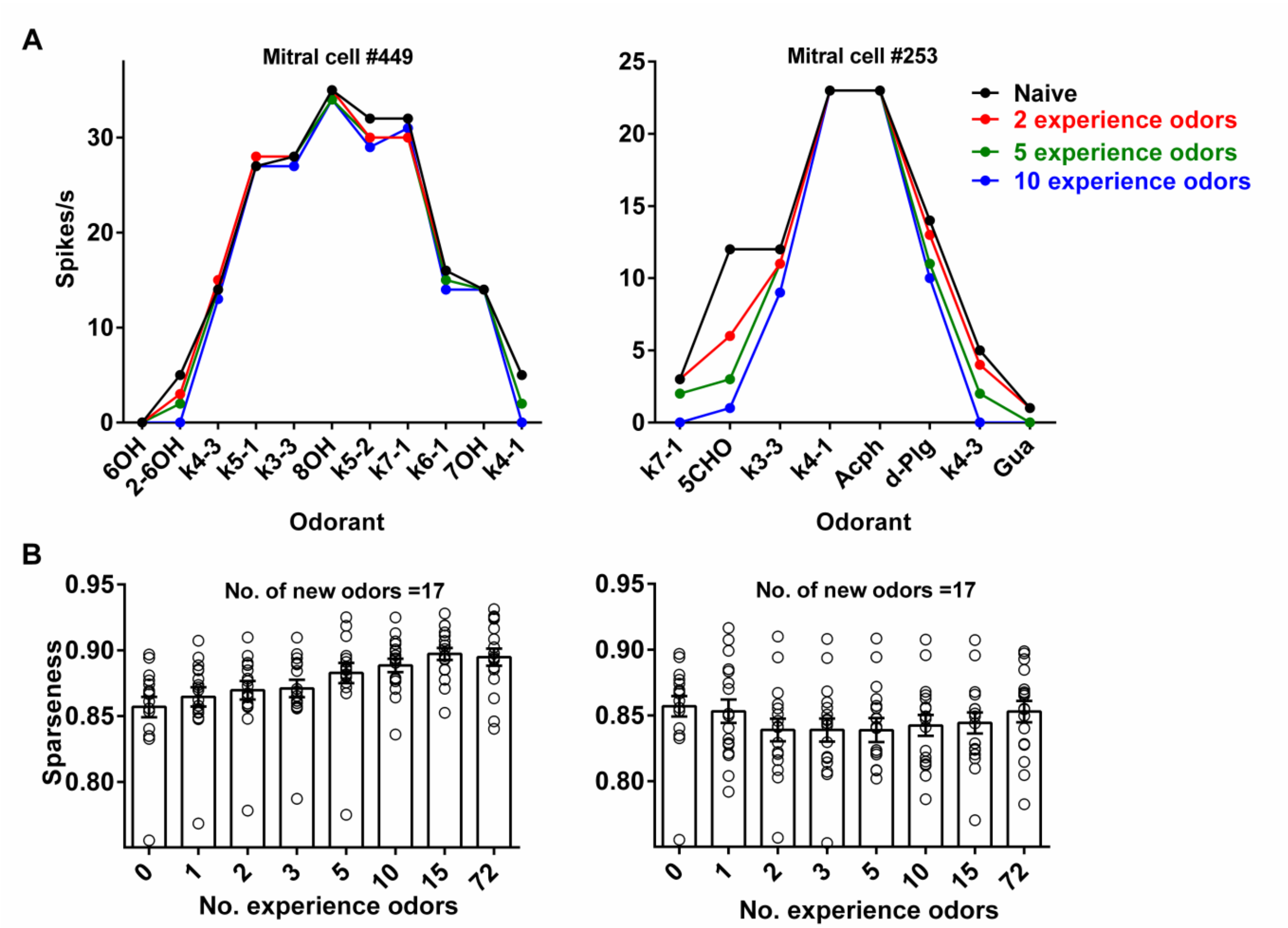

Supplementary Figure 5 Tuning specificity of mitral cells to new odors. (A) Responses of mitral cell 449 (left) and mitral cell 449 (right) to variety of odor inputs under naïve and 2, 5 and 10 experienced odors conditions. (B) Sparseness measured in last sniff period of the mitral cell network response to 17 new odor inputs in naïve and prior odor experience conditions (left, same as in Figure 2C). Responses of mitral cell receiving no input from a given new odor was set arbitrarily the same as that in the naïve condition and left the rest responses of mitral cells (receiving at least 1 intensity from new odor) unchanged as in Figure 2C.

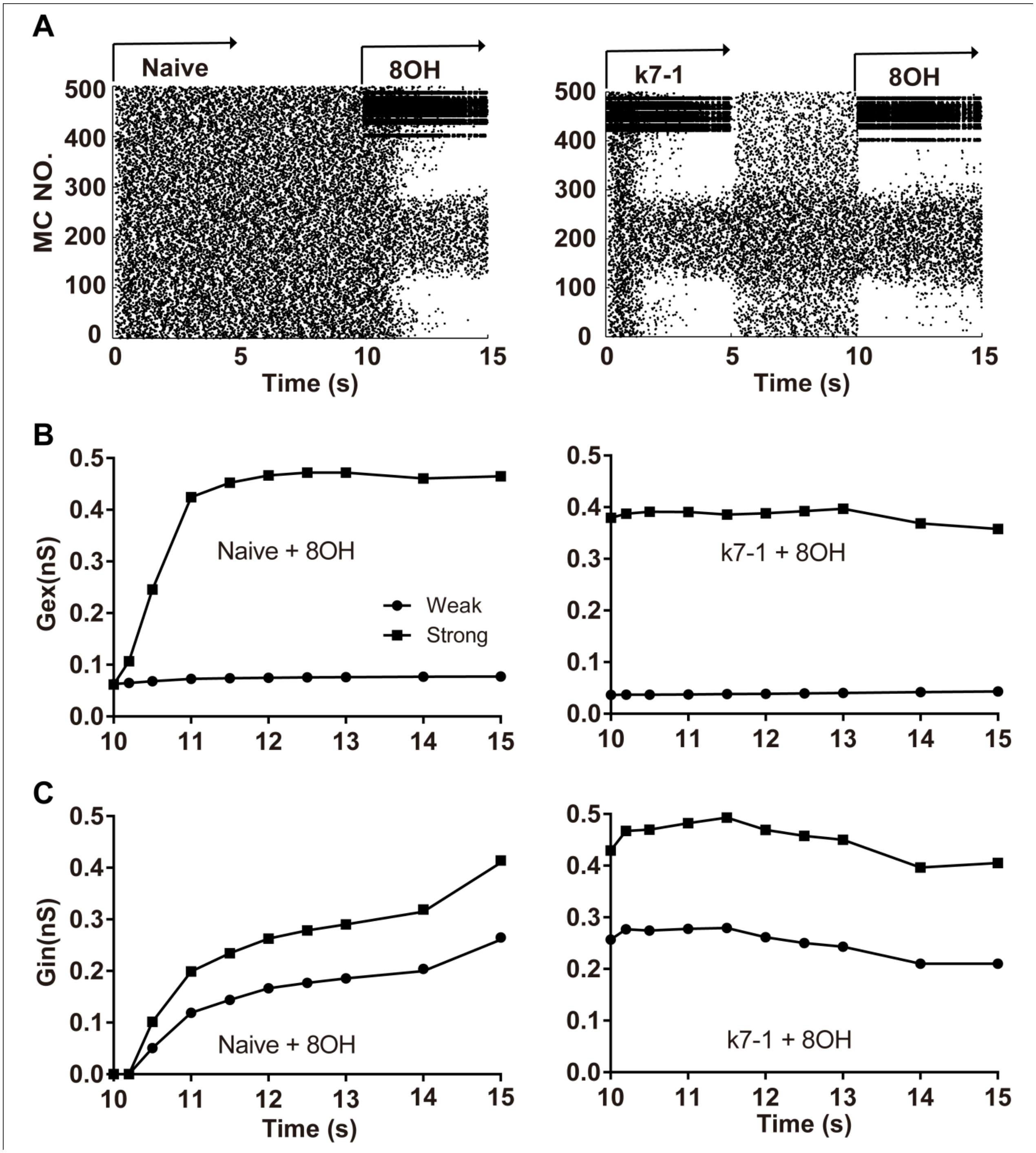

Supplementary Figure 6 Time course of synaptic weight of the mitral cell network to 8OH odor input. (A) Raster plot shows the mitral cell network response to 8OH in the naïve (left) or k7-1 experience (right) conditions. (B) Time evolution of the average excitatory (G_{ex}) weight of mitral cells receiving strong or weak input in network to 8OH input in naïve condition (left) or k7-1 experience (right). (C) Same as in (A) but shows the evolutions of the average inhibitory weight (G_{in}).
